# Genome-Wide Analysis of the Effects of Heat Shock on a *Saccharomyces cerevisiae* Mutant With a Constitutively Activated cAMP-Dependent Pathway

**DOI:** 10.1002/cfg.415

**Published:** 2004-07

**Authors:** Dawn L. Jones, June Petty, David C. Hoyle, Andrew Hayes, Stephen G. Oliver, Isabel Riba-Garcia, Simon J. Gaskell, Lubomira Stateva

**Affiliations:** 1 Department of Biomolecular Sciences UMIST PO Box 88 Manchester M60 1QD UK; 2 School of Biological Sciences University of Manchester The Michael Smith Building Oxford Road Manchester M13 9PT UK; 3 Department of Computer Science University of Manchester Oxford Road Manchester M13 9PT UK; 4 Michael Barber Centre for Mass Spectrometry Department of Chemistry UMIST PO Box 88 Manchester M60 1QD UK; 5 Department of Computer Science University of Exeter North Park Road Exeter EX4 4QF UK

## Abstract

We have used DNA microarray technology and 2-D gel electrophoresis combined with mass spectrometry to investigate the effects of a drastic heat shock from 30℃ to 50℃ on a genome-wide scale. This experimental condition is used to differentiate between wild-type cells and those with a constitutively active cAMP-dependent pathway in *Saccharomyces cerevisiae*. Whilst more than 50% of the former survive this shock, almost all of the latter lose viability. We compared the transcriptomes of the wildtype and a mutant strain deleted for the gene *PDE2*, encoding the high-affinity cAMP phosphodiesterase before and after heat shock treatment. We also compared the two heat-shocked samples with one another, allowing us to determine the changes that occur in the *pde2*Δ mutant which cause such a dramatic loss of viability after heat shock. Several genes involved in ergosterol biosynthesis and carbon source utilization had altered expression levels, suggesting that these processes might be potential factors in heat shock survival. These predictions and also the effect of the different phases of the cell cycle were confirmed by biochemical and phenotypic analyses. 146 genes of previously unknown function were identified amongst the genes with altered expression levels and deletion mutants in 13 of these genes were found to be highly sensitive to heat shock. Differences in response to heat shock were also observed at the level of the proteome, with a higher level of protein degradation in the mutant, as revealed by comparing 2-D gels of wild-type and mutant heat-shocked samples and mass spectrometry analysis of the differentially produced proteins.


**Comparative and Functional Genomics**
*Comp Funct Genom* 2004; **5**: 419–431.
Published online in Wiley InterScience (www.interscience.wiley.com). **DOI:** 10.1002/cfg.415

**Research Article**

### Genome-wide analysis of the effects of heat shock on a *Saccharomyces cerevisiae* mutant with a constitutively activated cAMP-dependent pathway

Dawn L. Jones[1], June Petty[2], David C. Hoyle[3#], Andrew Hayes[2], Stephen G. Oliver[2], Isabel Riba-Garcia[4], Simon J. Gaskell[4] and Lubomira Stateva[1]*

[1]*Department of Biomolecular Sciences, UMIST, PO Box 88, Manchester M60 1QD, UK*
[2]*School of Biological Sciences, University of Manchester, The Michael Smith Building, Oxford Road, Manchester M13 9PT, UK*
[3]*Department of Computer Science, University of Manchester, Oxford Road, Manchester M13 9PT, UK*
[4]*Michael Barber Centre for Mass Spectrometry, Department of Chemistry, UMIST, PO Box 88, Manchester M60 1QD, UK*

**Correspondence to:*
*Dr Lubomira Stateva, Department of Biomolecular Sciences, UMIST, PO Box 88, Manchester M60 1QD, UK.*
*E-mail: lubomira.stateva@umist.ac.uk*

*#Current address: Department of Computer Science, University of Exeter, North Park Road, Exeter EX4 4QF, UK..*

Received: 23 February 2004
Revised: 7 June 2004
Accepted: 17 June 2004

### Abstract

**We have used DNA microarray technology and 2-D gel electrophoresis combined with mass spectrometry to investigate the effects of a drastic heat shock from 30 °C to 50 °C on a genome-wide scale. This experimental condition is used to differentiate between wild-type cells and those with a constitutively active cAMP-dependent pathway in *Saccharomyces cerevisiae*. Whilst more than 50% of the former survive this shock, almost all of the latter lose viability. We compared the transcriptomes of the wild-type and a mutant strain deleted for the gene *PDE2*, encoding the high-affinity cAMP phosphodiesterase before and after heat shock treatment. We also compared the two heat-shocked samples with one another, allowing us to determine the changes that occur in the *pde2*Δ mutant which cause such a dramatic loss of viability after heat shock. Several genes involved in ergosterol biosynthesis and carbon source utilization had altered expression levels, suggesting that these processes might be potential factors in heat shock survival. These predictions and also the effect of the different phases of the cell cycle were confirmed by biochemical and phenotypic analyses. 146 genes of previously unknown function were identified amongst the genes with altered expression levels and deletion mutants in 13 of these genes were found to be highly sensitive to heat shock. Differences in response to heat shock were also observed at the level of the proteome, with a higher level of protein degradation in the mutant, as revealed by comparing 2-D gels of wild-type and mutant heat-shocked samples and mass spectrometry analysis of the differentially produced proteins. Copyright © 2004 John Wiley & Sons, Ltd.**

**Keywords:** ***Saccharomyces cerevisiae: PDE2*** **deletion; cAMP-dependent pathway; heat shock; transcriptome and proteome profiling**

### Introduction

The cAMP-mediated pathway is a highly conserved signal transduction pathway (Borges-Walmsley and Walmsley, 2000), which, in *Saccharomyces cerevisiae*, controls several processes including cell-cycle progression, cell growth and proliferation (Baroni *et al.*, 1994; Tokiwa *et al.*, 1994), reprogramming of transcription at the diauxic transition (Boy-Marcotte *et al.*, 1996), mating (Arkinstall *et al.*, 1991), pseudohyphal morphogenesis (Gimeno *et al.*, 1992; Pan and Heitman, 1999) and metabolism and stress responses (Broach, 1991; Thevelein and Winde, 1999; Thevelein *et al.*,

Copyright © 2004 John Wiley & Sons, Ltd.

2000). It operates via the second messenger cAMP, the only known biochemical role of which is to activate protein kinase A (PKA) (Cannon and Tatchell, 1987; Toda *et al.*, 1987a, 1987b). High activity of PKA in yeast leads to low levels of the storage carbohydrates trehalose and glycogen, low stress resistance due to reduced expression of STRE (stress response element)-controlled genes, aberrant $G_0$ arrest, poor growth on non-fermentable and weakly fermentable carbon sources and failure of sporulation in diploid cells. Low activity yields the opposite phenotypes, with derepression of STRE-controlled genes leading to high stress resistance, constitutive expression of heat-shock genes, and sporulation of diploid cells in rich media (Broach and Deschenes, 1990; Thevelein, 1992, 1994; Tatchell, 1993; Ruis and Schuller, 1995). Two *trans*-acting factors (Msn2p and Msn4p), negatively regulated by PKA, have been shown to be involved in STRE-mediated gene expression (Martinez-Pastor *et al.*, 1996; Schmitt and McEntee, 1996).

Intracellular levels of cAMP are controlled by cAMP-phosphodiesterases, which catalyse the degradation of cAMP. The genome of *S. cerevisiae* contains two genes encoding cAMP-phosphodiesterases (PDEs) *PDE1* and *PDE2* (Sass *et al.*, 1986; Nikawa *et al.*, 1987). Pde1p, the low cAMP-affinity phosphodiesterase, is an enzyme with $K_m$ value of 20–250 μM, which plays a role in controlling glucose and intracellular acidification-induced cAMP signalling (Ma *et al.*, 1999). The high-affinity cAMP phosphodiesterase Pde2p is an $Mg^{2+}$-requiring, zinc-binding enzyme with a $K_m$ for cAMP of 170 nM (Suoranta and Londesborough, 1984; Sass *et al.*, 1986; Wilson and Tatchell, 1988), which controls the basal cAMP levels in the cell and protects it from interference by extracellular cAMP (Wilson *et al.*, 1993).

Mutants with a constitutively activated cAMP-dependent pathway, especially those resulting from the deletion of *PDE2*, have been instrumental in determining the numerous roles of cAMP in budding yeast. Recently, we showed that deletion of *PDE2* affected a significant number of cell wall genes, including several of previously unknown function, causing a range of cell wall defects (Jones *et al.*, 2003). Previously it has been reported that a deletion of *PDE2* reduced the ability of yeast cells to survive stress conditions such as heat shock and nitrogen starvation (Sass *et al.*, 1986; Wilson and Tatchell, 1988). When eukaryotic cells are subjected to heat shock, they respond by activating the expression of heat shock genes, utilizing the Msn2p/Msn4p and heat shock transcription factors (Xiao and Lis, 1988; Boorstein and Craig, 1990). DNA microarrays have been extensively used in experiments investigating responses to environmental changes (Gasch *et al.*, 2000, 2001; Causton *et al.*, 2001), including mild heat treatment at 37 °C. We have used this technology to determine the changes in the cellular transcriptome after a more drastic heat shock from 30 °C to 50 °C. This experimental condition differentiates between wild-type cells and those with a constitutively activated cAMP pathway, as the former survive, whilst cells of the latter lose almost all viability upon this type of shock (Toda *et al.*, 1985). We investigated the expression profiles of the wild-type and the *pde2*Δ mutant strain compared to their respective heat-shocked samples; we also looked at how the two heat-shocked samples compared with one another, allowing us to establish which transcription pattern determines loss of viability in the *pde2*Δ mutant. The results of the microarray analysis suggested that ergosterol biosynthesis and carbon source utilization might be potential factors contributing to heat shock survival. These predictions were confirmed by biochemical and phenotypic tests. Of 146 orphan genes (yeast genes of previously unknown function) identified amongst the genes with altered expression levels, 13 were found to be linked to the cAMP pathway, as suggested by the high heat-shock sensitivity phenotype of their respective deletion mutants. The different response to heat shock was manifested at the proteome level as well as revealed by classical proteome analysis using 2-D gel electrophoresis and subsequent mass spectrometry analysis of the majority of the differentially produced proteins. However, there was little if any correlation between transcriptome and proteome expression data.

### Methods

### Strains and media

*Saccharomyces cerevisiae* FY23 (Winston *et al.*, 1995) was used as the reference wild-type strain and as the host for the construction of the *pde2*Δ mutant DJ28, as previously described (Jones *et al.*,

Copyright © 2004 John Wiley & Sons, Ltd. 

2003). BY4741 (*MAT***a** *his3*Δ1 *leu2*Δ0 *met15*Δ0 *ura3*Δ0) (ATCC 4040002) was the parental isogenic strain of the mutants in heat-shock-specific genes of previously unknown function, identified in the present study. YPD was used routinely throughout (Sherman *et al.*, 1986).

### Continuous culture conditions

Wild-type and mutant strains were grown in YPD in continuous culture at 30 °C in 2 l fermentors (Applikon Biotechnology) with a 1 l working volume at a dilution rate of 0.1/h. The stirring speed was 750 rpm with an air-flow of 1 l/min. The pH was automatically controlled at 4.5 by the addition of 2.5 M NaOH. Samples were heat shocked at 50 °C for 10 min after control samples were removed from the fermentors. All samples were frozen in liquid nitrogen and stored at −80 °C for further analysis.

### Viability and budding index

Viability was calculated as the percentage of cells (CFU) that survived the heat shock compared to the control plate of non-stressed cells. A synchronous culture was set up by addition of 300 μg/ml alpha mating factor (Sigma). Once 95% of cells had arrested in $G_1$, cells were released from pheromone arrest by washing three times with water before resuspension in YPD media. These cells were then heat shocked for 10 min at 50 °C at different stages of the cell cycle. The budding index was calculated as the number of cells on which buds could be seen.

### Ergosterol test

Percentage ergosterol, in the cells obtained from the chemostat cultures, was determined as described (Arthington-Skaggs *et al.*, 1999).

### RNA extraction, cDNA synthesis and RT-PCR

These conditions were essentially as previously described (Jones *et al.*, 2003). Briefly, total RNA was extracted using TriZOL reagent (Invitrogen), contaminating DNA was removed by dilution of the RNA sample followed by treatment with DNase I for 30 min at 37 °C, the RNA was then LiCl-precipitated and resuspended in RNase-free water. Copy DNA (cDNA) was prepared using 0.5 μg oligo dT and Superscript II (both Invitrogen). RT-PCR conditions were 95 °C for 1 min, 51 °C for 1 min, and 72 °C for 2 min (30 cycles). Control reactions using RNA, as a template, in the PCR ensured that no contaminating DNA was present in the sample.

### RNA extraction, cDNA synthesis and labelling for microarray analysis

These conditions were essentially as previously described (Jones *et al.*, 2003). Briefly, cells were frozen in liquid nitrogen and stored at −80 °C until RNA was prepared (Hauser *et al.*, 1998). Frozen cells were transferred to a pre-cooled Teflon vessel containing a 7 mm tungsten carbide bead and placed into a Micro-Dismembrator (B. Braun Biotech). Total RNA was extracted using TriZOL reagent (Invitrogen) according to the manufacturer's instructions. Single-stranded nucleic acids were precipitated with LiCl and resuspended in DEPC-treated water before synthesis and labelling of cDNA (Hegde *et al.*, 2000). Labelling efficiency was assessed by visualization on a Storm 860 phosphoimager (Amersham) following agarose gel electrophoresis.

### Microarray hybridization and image analysis

Microarrays based on PCR products of all *S. cerevisiae* open reading frames were manufactured in-house in the Transcriptome Resource Facility of the Consortium for the Functional Genomics of Microbial Eukaryotes (COGEME). The differentially labelled samples were hybridized to the array slides overnight at 42 °C before being washed as described previously (Jones *et al.*, 2003). All washes were carried out in Coplin jars in the dark at room temperature with agitation. The slides were dried by centrifugation at 3000 rpm. The hybridized arrays were scanned with a GenePix 4000A scanner (Axon Instruments). Artefacts were removed after visual inspection of the spots and the intensity of fluorescence adjusted to the medians.

### Microarray data analysis and quality control

To minimize the effect the intrinsic variability involved in this technique has upon the estimates of differential expression, a minimum of

Copyright © 2004 John Wiley & Sons, Ltd. 

eight microarray slides were used for each experiment. These were technical repeats formed by taking aliquots from a single chemostat culture for each experimental condition. Half of the slides involved reciprocal dye labelling, allowing compensation for the differences in preferential incorporation of the two fluorescent labels and their responses to be corrected for statistically. The raw data can be found on the COGEME website: **http://www.cogeme.man.ac.uk/DawnsData/Data2/**

Spot selection and normalization of the raw spot intensities were essentially as previously described (Jones *et al.*, 2003). Briefly, in order to obtain an accurate indication of the proportional changes of gene expression in the mutant compared to the wild-type cells, the biases must be removed from the raw data before useful biological information can be extracted.

The error model given in the equation below expresses the biases that we can remove and we consider to be present in the raw log-ratio $M_{ij}$ for the $i$th probe in the $j$th hybridization:

$$M_{ij} = x_i + b_0 + b_j + f_j(A_{ij}) + e_j(P_i) + e(S_i) + \varepsilon_{\mathrm{ij}}$$

where $b_0$ and $b_j$ represent global (array-wide) biases present in every measured log-ratio. $f_j(A_{ij})$ represents a bias dependent on the average log intensity $A_{ij}$ (Dudoit *et al.*, 2002; Yang *et al.*, 2002). $e_j(P_i)$ and $e(S_i)$ represent spatial and probe-dependent biases, respectively, and the residual error is denoted by $\varepsilon_{\mathrm{ij}}$. We take the remaining residual error $\varepsilon_{ij}$ to be dominated by non-systematic experimental error that is characterized only by the particular hybridization $j$. After removal of the biases, we obtain an estimate $\hat{x}_i$ for the true log-ratio $x_i$ of probe $i$. Critical values of $\hat{x}_i$ for each probe, at the 1% significance level ($p < 1\%$), are calculated by re-sampling from the residuals of each hybridization. From this we can identify those genes that we believe to be genuinely differentially expressed between the two labelled populations of mRNA.

### 2-D polyacrylamide gel electrophoresis

### Sample preparation and 1st dimension

Cells obtained from the chemostat cultures were counted using a haemocytometer and $1-2 \times 10^9$ cells were transferred to a tube containing Red Matrix™ (Bio101) before being pelleted in a centrifuge. The pellet was resuspended in 160 µl 10 mM Tris (pH 7.5) containing PMSF, pepstatin A, and complete protease inhibitor cocktail with EDTA (Roche), DNase 1 and RNase and bead-beaten in a Mini-Beadbeater 8 at full speed for 5 min. Cell lysis was confirmed under a light microscope. Rehydration solution (8 M urea, 2 M thiourea, 40 mM Tris, 2% (w/v) CHAPS, 1% (v/v) Igepal, 2% (v/v) IPG buffer, 10 mM DTT and bromophenol blue) was then added to the cell lysate and incubated at room temperature for 4–5 h with occasional vortexing. The solution was cleared of cellular debris by brief centrifugation and 450 µl cleared solution was added to each 18 cm Immobiline pH 3-10 NL DryStrip. DryStrips were rehydrated overnight before being focused on an IPGphor Isoelectric Focusing System (Pharmacia) for 80 000 Vh.

### 2nd Dimension

DryStrips were equilibrated according to the manufacturers' instructions prior to loading on a vertical 12% SDS-polyacrylamide gel and 2nd dimension electrophoresis was carried out according to the manufacturers' instructions. Proteins were visualized by staining with colloidal Coomassie (Sigma) according to the manufacturer's instructions. Consistent spot differences were identified (between at least four independently run gels) and the corresponding spots excised from the gel for mass spectrophotometric analysis. Our definition of a difference in gels was not a quantitative measure of the protein; rather, it was a protein being present or absent from the gel.

### Mass spectrometry

Mass spectrometry analysis was performed at the Proteome Resource Facility (PRF-2) of COGEME and was essentially as previously described (Shevchenko *et al.*, 1996). Briefly, excised spots were incubated in 200 µl 200 mM ammonium acetate solution for 20 min and dehydrated in acetonitrile for 20 min. This process was repeated twice before the spots were dried on a speed vacuum centrifuge. In-gel digestion of the excised proteins was carried out by adding 5–10 µl 7.5 ng/µl trypsin solution in 50 mM ammonium acetate to the gel pieces, and the samples were

Copyright © 2004 John Wiley & Sons, Ltd. 

incubated overnight at 37 °C. Supernatants were then removed and 50 μl acetonitrile was added to the gel pieces; after 10 min the supernatants were pooled together and dried on a speed vacuum centrifuge. Samples were desalted using Zip-Tip (Millipore) (following the manufacturer's instructions) and 1 μl desalted sample was mixed with 1 μl matrix and spotted onto the MALDI target. Samples were air-dried at room temperature and analysed using α-cyano-4-hydroxycinnamic acid as the matrix. A Voyager-DE STR (Applied Biosystems, Franingham, MA, USA) was used to generate spectra (the average of 100 shots). The accelerating voltage was 20 000 V, the delay 235 ns and the grid voltage 76%. An external seven-point calibration was used. For digested samples analysed by tandem mass spectrometry, product ion analyses were performed on a Q-ToF I (Waters/Micromass Ltd, Wythenshawe, Manchester, UK). Samples were introduced by glass capillaries, capillary voltage was set at 800 V, cone voltage was 45 V, the set collision voltage was 29 V and argon was used as collision gas at a recorded pressure of $5 \times 10^{-5}$ mBar. Data acquisition and processing were controlled via the Masslynx data system.

### Results

### Heat shock sensitivity is cell cycle-dependent

DJ28, a previously generated *pde2*Δ mutant isogenic with the wild-type reference strain FY23 (Winston *et al.*, 1995) was used in this study, so that our transcriptome data could be added to the pre-existing transcriptome data generated in the reference genetic background. One of the phenotypic characteristics of this *pde2*Δ mutant and, indeed, any other yeast mutant with a constitutively activated cAMP-dependent pathway, is inability to survive heat shock (Sass *et al.*, 1986; Toda *et al.*, 1985). A quantitative viability study revealed the severity of this phenotype (Figure 1). Only 1–2% of the *pde2*Δ cells were viable after heat shock, compared to 50% of the wild-type cells. Since oscillating cAMP levels have been reported to occur during the cell-cycle of both glucose limited and glucose repressed synchronized cultures (Muller *et al.*, 2003), we decided to test the theory that only 50% of the wild-type cells survived because of a varying susceptibility to heat shock of cells in the different stages of the cell cycle due to these cAMP-level oscillations. When an α-factor synchronized cell culture of the wild-type strain was subjected to the heat shock (at 30 min intervals from α-factor release), budding cells were much more sensitive than those in $G_1$, which could explain the reduced viability of the wild-type strain whose growth was not synchronized in the chemostat culture used (Figure 2).

### Different transcriptome profiles were revealed for wild-type and *pde2*Δ strains after heat shock

Three comparisons were carried out in this study on the effects of heat shock in *S. cerevisiae*. First, the changes in the wild-type's transcriptome due to heat shock from 30 °C to 50 °C were investigated to characterize the normal cellular response. Second, changes during the heat shock were identified in the *pde2*Δ mutant, which were expected to represent the effects of a constitutively activated cAMP-dependent pathway on heat shock survival. Finally, comparing heat-shocked wild-type to heat-shocked mutant sample constituted analysis of the end-points. The raw data can be found on **http://www.cogeme.man.ac.uk/DawnsData/Data2/**. After applying the normalization algorithms described in Materials, the genes with

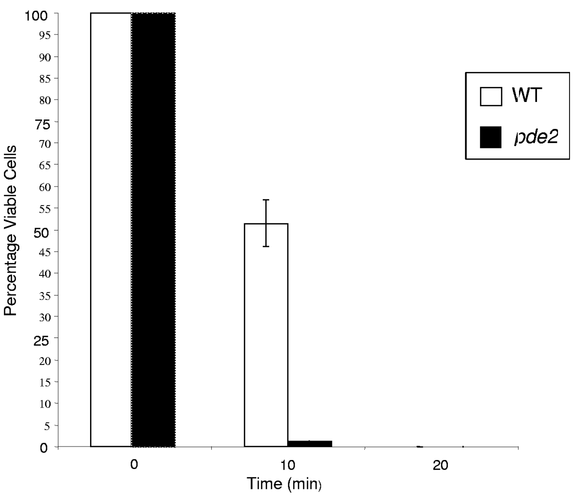

**Figure 1.** Viability of FY23 (WT) and DJ28 (*pde2*Δ) after heat shock. Viability was calculated as the percentage of cells (CFU) that survived the heat shock at 50 °C for 10 min compared to the control of non-stressed cells. Each calculation was done in triplicate

Copyright © 2004 John Wiley & Sons, Ltd.

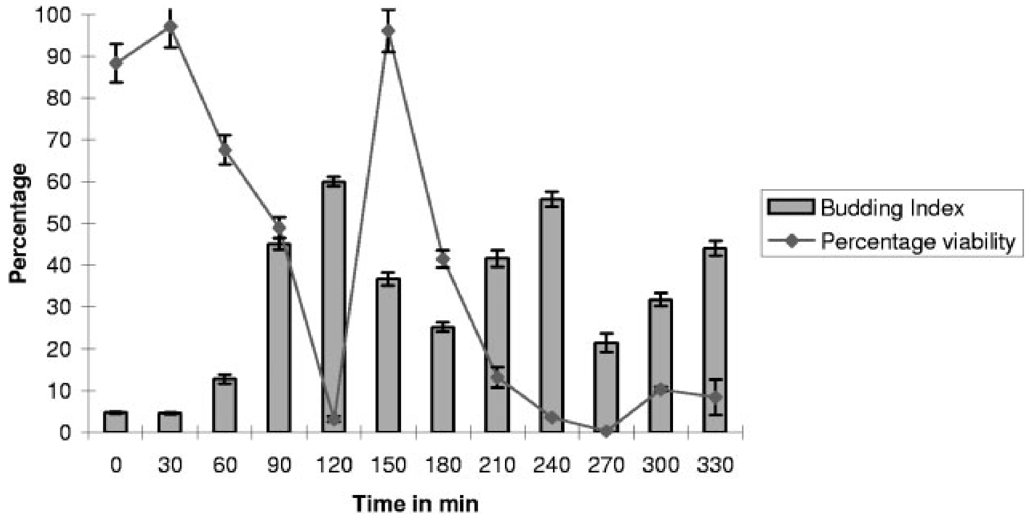

**Figure 2.** Effect of cell cycle phase on heat shock survival of FY23 (WT). Heat shock was at 50 °C for 10 min. Viability was calculated as the percentage of cells (CFU) that survived the heat shock compared to the control. Each calculation was done in triplicate. Synchronization of the culture was achieved by treatment with 300 μg/ml $\alpha$ mating factor (Sigma). Samples were taken from the synchronized culture after release from the pheromone arrest at indicated time points and subjected to the heat shock

**Table 1.** Functional categorization of differentially expressed genes identified in the different comparisons

| MIPS functional category | Percentage of differentially upregulated genes | | | Percentage of differentially downregulated genes | | |
|---|---|---|---|---|---|---|
| | **WT/WT$^{HS}$** | ***pde2/pde2*$^{HS}$** | **WT$^{HS}$/*pde2*$^{HS}$** | **WT/WT$^{HS}$** | ***pde2/pde2*$^{HS}$** | **WT$^{HS}$/*pde2*$^{HS}$** |
| Metabolism | 7.4 | 14 | 9.5 | 11.1 | 16.2 | 16.2 |
| Energy | 0 | 2.3 | 1.2 | 3.1 | 4.6 | 6.9 |
| Cell growth | 7.4 | 0 | 6.5 | 7.6 | 5.7 | 3.8 |
| Transcription | 1.9 | 4.7 | 7.7 | 0 | 3.6 | 1.5 |
| Protein synthesis | 0 | 0 | 9.1 | 5.1 | 1.5 | 0.8 |
| Protein destination | 7.4 | 4.7 | 4.9 | 6 | 5.1 | 6.2 |
| Transport facilitation | 5.6 | 7 | 2.6 | 2.3 | 6.4 | 4.6 |
| Cellular transport | 1.9 | 4.7 | 4.6 | 4.8 | 8 | 3.8 |
| Cellular biogenesis | 0 | 2.3 | 1.5 | 1.8 | 1.5 | 0.8 |
| Signal transduction | 0 | 0 | 0.7 | 1.5 | 1.5 | 1.5 |
| Cell rescue | 9.3 | 4.7 | 2.2 | 3.1 | 2.2 | 13.1 |
| Ionic homeostasis | 0 | 0 | 0.9 | 1.1 | 1.3 | 0.8 |
| Cellular organization | 13 | 18.6 | 25.4 | 23.9 | 21.7 | 21.5 |
| Classification not clear | 1.9 | 2.3 | 1.1 | 1.3 | 1.3 | 0 |
| Transposable elements | 0 | 0 | 0 | 0.1 | 0 | 0 |
| Unknown | 44.4 | 34.9 | 22 | 20.4 | 19.5 | 18.5 |
| Total number of differentially expressed genes (% genome) | 39 (0.64) | 28 (0.46) | 955 (15.66) | 2465 (40.41) | 311 (5.10) | 63 (1.03) |

altered expression levels were categorized according to the Munich Information Centre for Protein Sequences (MIPS) (Table 1). However, it should be emphasized that MIPS assign some of the genes to more than one category and this is reflected in the distribution. A significantly higher proportion of the cell rescue category genes were upregulated (9.3%) after heat shock in the wild-type compared to that of the *pde2*Δ mutant (4.7%); perhaps more significantly, when comparing the two

Copyright © 2004 John Wiley & Sons, Ltd. 

end-point experiments the *pde2*Δ mutant shows a downregulation of the cell rescue genes of 13.1% compared to the wild-type after heat shock. The protein synthesis category shows a downregulation in the wild-type after heat shock of 5.1% compared to 1.5% in the mutant. The metabolism category includes a higher percentage of upregulated genes in the mutant (14%) in comparison to the wild-type (7.4%). Likewise, there was an increased percentage of downregulated genes in this category (16.2%) in the mutant in comparison to the wild-type (11%).

Quantitative RT-PCR was used as an alternative method to validate the transcription levels of some of the differentially expressed genes identified by the microarray experiment. There was generally good agreement between the microarray data and the qRT-PCR data (Table 2).

### The transcriptome analysis reveals a set of heat shock survival genes

In order to characterize the factors involved in the mutant's response that determine inability to survive, a subset of genes was identified which corresponds to those genes specific to the heat shock response in the *pde2*Δ mutant. By comparing the three sets of data generated in the present study and the dataset obtained previously (Jones *et al.*, 2003), 421 genes specific to the mutant's response were identified (Table 1, Supplementary data). Differentially expressed genes that were common to both WT-WT$^{HS}$ and *pde2*Δ-*pde2*Δ$^{HS}$ were eliminated on the basis of being representatives of a heat-shock-specific response, unaffected by cAMP levels. Genes previously identified as *pde2*Δ-mutant-specific (Jones *et al.*, 2003) were also eliminated. Therefore, any genes that were found in the WT$^{HS}$-*pde2*Δ$^{HS}$ (all genes involved in the wild-type heat-shock response have already been eliminated as stated above) or *pde2*Δ-*pde2*Δ$^{HS}$, which could not be discounted using the other datasets, were deemed *pde2*Δ mutant heat-shock-specific genes with altered expression. The functional categorization of these genes according to the MIPS functional classification is shown in Figure 3. Almost all functional categories are represented. There is however, a considerably lower contribution of genes with unknown function in comparison to the previously reported cAMP-responsive genes (Jones *et al.*, 2003).

**Table 2.** Microarray expression data for several genes confirmed by qRT-PCR

| Data set/ condition | Gene | Expression* microarray | Expression qRT–PCR | Standard deviation RT-PCR |
|---|---|---|---|---|
| WT-WT$^{HS}$ | *ICY2* | 5.65 | 5.34 | ±0.91 |
| | *HSP42* | 3.12 | 3.53 | ±0.33 |
| | *BTN2* | 6.70 | 2.43 | ±0.06 |
| *pde2*Δ-*pde2*Δ$^{HS}$ | *COX6* | 4.31 | 2.31 | ±0.33 |
| WT$^{HS}$-*pde2*Δ$^{HS}$ | *FIS1* | 3.31 | 2.09 | ±0.08 |
| | *ICY2* | −5.15 | −1.69 | ±0.27 |
| | *HSP42* | −4.72 | −7.03 | ±0.89 |
| | *BTN2* | −6.00 | −2.91 | ±0.46 |

* Here and in all other tables 'Expression' represents the fold change in gene expression levels when the 'X' transcriptome is compared to the 'Y' transcriptome (X–Y). Negative represents downregulation whereas positive represents upregulation in 'X' compared to 'Y'.

### The extent of heat shock sensitivity is dependent on the carbon source in the medium

Eleven of the 18 (Andre, 1995; Kruckeberg, 1996; Nelissen *et al.*, 1997) hexose transporter genes are downregulated in the mutant heat-shock-specific subset of differentially expressed genes. Table 3 shows that *GAL2* and *MAL11* are also differentially downregulated. *HWT1–4, HXT6* and *HXT7* are the major hexose transporters in yeast transporting glucose, mannose and fructose (Reifenberger *et al.*, 1995, 1997). These results suggested that the carbon source might have an effect on survival after heat shock. We tested this hypothesis by determining the effect of different carbon sources on the viability of both wild-type and mutant cells after heat shock. There was some variation in the survival of the wild-type strain grown in the presence of different carbon sources in the medium, but only in the case of galactose was it considerably lower than the average of 50% (Figure 4). In contrast, the survival of the mutant cells varied considerably depending on the carbon source used, ranging from 2–60%. It was equal to or even higher than that of the wild-type after growth on mannose and galactose, respectively.

### Deletion of several orphan genes results in heat shock sensitivity

Of the 421 mutant heat-shock-specific differentially expressed genes, 146 (34.7%) were of previously unknown function (orphans). The possible role of these orphans in the cAMP-dependent

Copyright © 2004 John Wiley & Sons, Ltd.

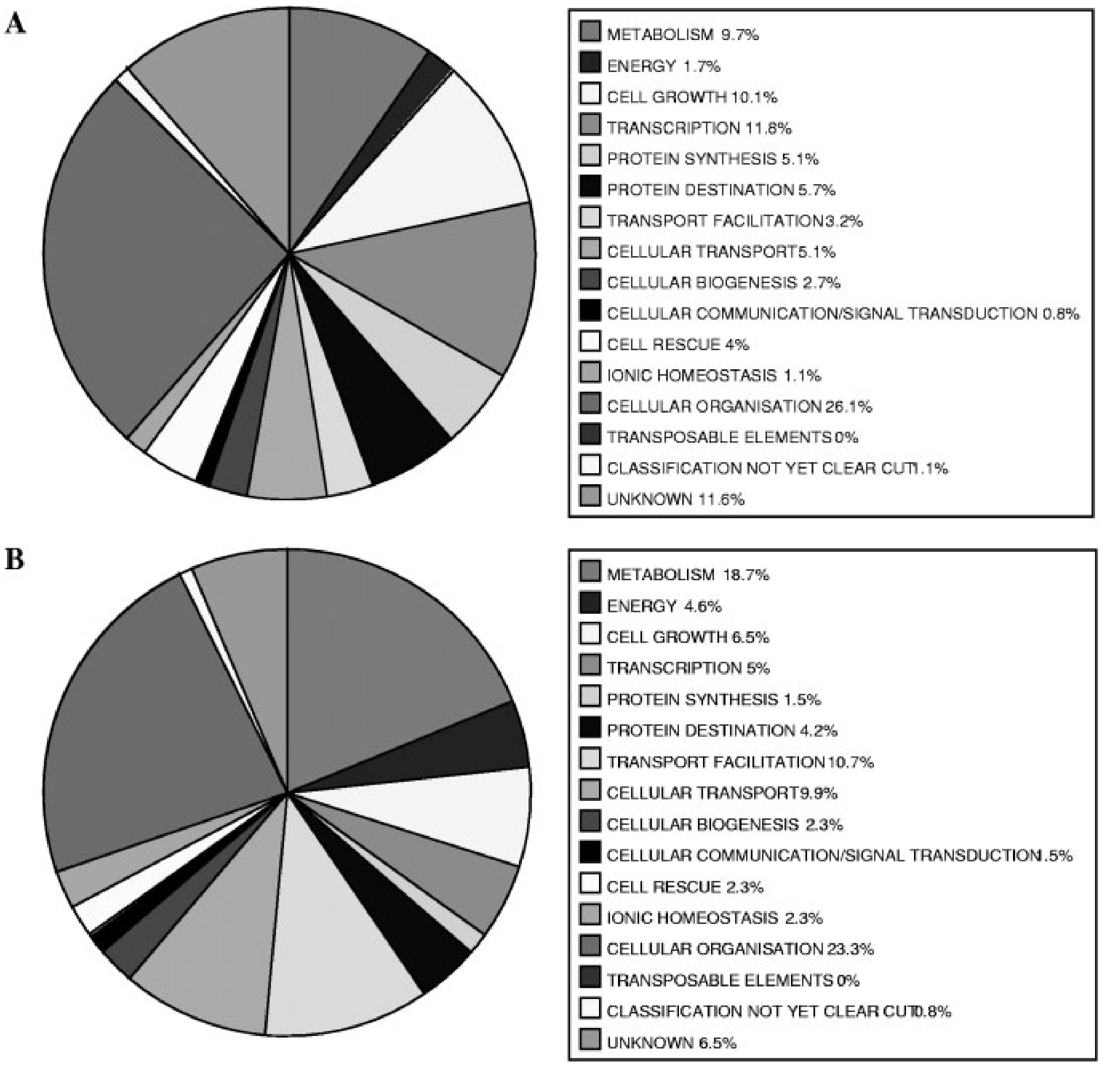

**Figure 3.** Distribution of *pde2*Δ mutant heat shock-specific differentially expressed genes according to MIPS functional categories. Differentially expressed genes in the *pde2*Δ mutant are divided into upregulated (A) and downregulated (B) genes. Some of the genes are assigned to more than one functional category, and this is reflected in the distribution pathway was investigated by testing the sensitivity to heat shock of their respective deletion mutants in the BY4741 background. The results of the primary screen can be found in Table 2 of the Supplementary data on the COGEME website: **http://www.cogeme.man.ac.uk/DawnsData/Data2/**. The second round of screening (Table 4) shows a different degree of sensitivity to heat shock displayed by the deletion mutants in 13 of these orphans. There have been previous reports that YBR266C is involved in endocytosis (Wiederkehr *et al.*, 2001); YLR 320W/*MMS22* is involved in double-strand break repair (Bennet *et al.*, 2001); YCR059C/*YIH1* is involved in regulation of amino acid metabolism (Kubota *et al.*, 2000); YGR221C/*TOS2*, is localized on the bud neck, bud tip (Drees *et al.*, 2001) and the deletion mutant in YLR052W/*IES3* exhibits growth defects on fermentable carbon source (Steinmetz *et al.*, 2002). For the rest of the orphans (YDR525W-A, YKR049C, YBR226C, YOR376W, YER071C, YHR080C, YLR169W and YOR131C) there are no data available in the database. It would appear that, apart from the reported viability of their respective

Copyright © 2004 John Wiley & Sons, Ltd. 

**Table 3.** Differentially expressed mutant heat-shock-specific genes involved in hexose transport

| Gene | Function | Expression |
|---|---|---|
| *HXT2* | High-affinity hexose transporter | −1.50 |
| *HXT3* | Low-affinity hexose transporter | −1.59 |
| *HXT4* | Moderate- to low-affinity glucose transporter | −1.53 |
| *HXT6* | High-affinity hexose transporter | −1.68 |
| *HXT7* | High-affinity hexose transporter | −1.62 |
| *HXT8* | Hexose transport protein | −1.53 |
| *HXT9* | Hexose transport protein | −1.44 |
| *HXT10* | Hexose transporter | −1.72 |
| *HXT11* | Low-affinity glucose transport protein | −1.48 |
| *HXT12* | Strong similarity to sugar transport proteins | −1.63 |
| *HXT17* | Sugar transport protein | −1.62 |
| *GAL2* | Galactose (and glucose) permease | −1.59 |
| *MAL11* | General α-glucoside permease | −1.62 |

**Table 4.** Heat shock sensitivity of deletion mutants in orphan genes

| ORF | Expression | Survival level# | Identified in data set/condition |
|---|---|---|---|
| BY4741 (wt) | | ++++++ | |
| YOR376W | 1.41 | +++ | $WT^{HS}/pde2\Delta^{HS}$ |
| YBR266C | 1.82 | +++++ | $WT^{HS}/pde2\Delta^{HS}$ |
| YER071C | 1.42 | +++++ | $WT^{HS}/pde2\Delta^{HS}$ |
| YHR080C | 1.68 | ++++ | $WT^{HS}/pde2\Delta^{HS}$ |
| YLR169W | −1.57 | ++++ | $WT/WT^{HS}$ |
| YOR131C | −1.60 | +++ | $pde2\Delta/pde2\Delta^{HS}$ |
| YLR320W | 1.65 | +++++ | $WT^{HS}/pde2\Delta^{HS}$ |
| YDR525W-A | −1.66 | +++ | $pde2\Delta/pde2\Delta^{HS}$ |
| YGR221C | −1.56 | ++++ | $pde2\Delta/pde2\Delta^{HS}$ |
| YGL050W | 1.63 | +++ | $WT^{HS}/pde2\Delta^{HS}$ |
| YKR049C | 1.30 | +++ | $WT^{HS}/pde2\Delta^{HS}$ |
| YLR052W | −1.89 | +++ | $pde2\Delta/pde2\Delta^{HS}$ |
| YCR059C | 1.35 | ++ | $WT^{HS}/pde2\Delta^{HS}$ |

### Heat shock condition was 10 min at 50 °C. Survival is represented as follows:
++++++ growth at all dilutions ($10^7$–$10^2$).
+++++ growth at dilutions down to $10^3$.
++++ growth at dilutions down to $10^4$.
+++ growth at dilutions down to $10^5$.
++ growth at dilutions down to $10^6$.

systematic deletion mutants (Giaever *et al.*, 2002), our observation of heat shock sensitivity is the first phenotype ascribed to these mutants.

### Ergosterol biosynthesis contributes to heat shock sensitivity

A large number of genes involved in ergosterol biosynthesis were amongst the differentially expressed genes (Table 5). Alteration in the expression of these genes could be observed in all of the comparisons made. The microarray data would predict that there would be differences in the ergosterol contents of the wild-type and the mutant, which could be a contributing factor to their different levels of survival after heat shock. A relationship between ergosterol content and resistance to heat stress has been observed previously (Swan and Watson 1998). In order to test this microarray data prediction, we measured the percentage of ergosterol in each of the cell samples before

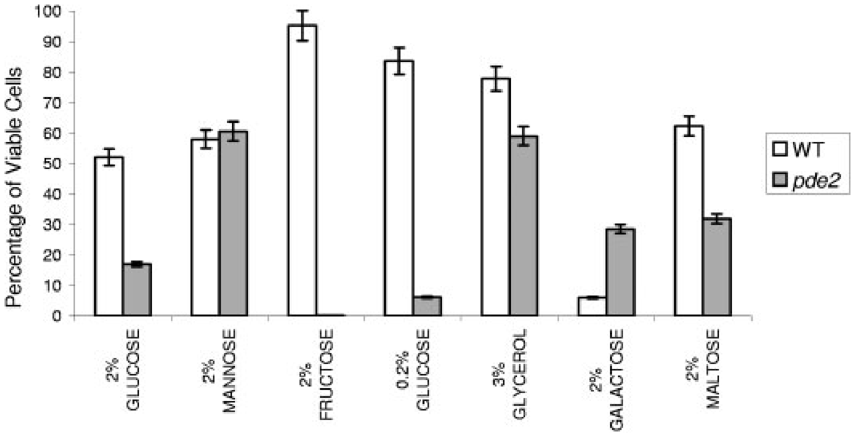

**Figure 4.** Effect of carbon sources on heat shock survival. FY23 (WT) and DJ28 (*pde2*Δ) were grown on YP supplemented with different carbon sources and their survival after heat shock at 50 °C for 10 min was determined as the percentage of cells (CFU) that survived the heat compared to the control. Each calculation was done in triplicate

Copyright © 2004 John Wiley & Sons, Ltd. 

and after heat shock. The percentage of ergosterol was slightly reduced in the *pde2*Δ mutant before heat shock, but was almost double that found in the wild-type after heat shock (Table 6).

**Table 5.** Differential expression of ergosterol encoding genes across all four comparisons

| Data set/condition | Gene | Expression |
|---|---|---|
| WT-*pde2*Δ | *ERG6* | −1.4 |
| | *ERG10* | 1.28 |
| | *ERG3* | 1.26 |
| WT-$WT^{HS}$ | *ERG9* | −1.8 |
| | *ERG8* | −1.79 |
| | *ERG27* | −1.54 |
| | *ERG26* | −1.61 |
| | *ERG20* | −1.81 |
| | *ERG2* | −1.5 |
| | *ERG13* | −1.56 |
| | *ERG12* | −1.52 |
| | *ERG11* | −1.59 |
| | *ERG10* | −1.42 |
| *pde2*Δ-*pde2*$\Delta^{HS}$ | *ERG3* | −1.42 |
| | *ERG24* | −1.41 |
| $WT^{HS}$-*pde2*$\Delta^{HS}$ | *ERG10* | 1.58 |
| | *ERG2* | 1.82 |
| | *ERG27* | 1.44 |
| | *ERG7* | 1.56 |

**Table 6.** Percentage ergosterol content

| Strain/condition | Ergosterol (%) | SD | SEM |
|---|---|---|---|
| WT | 1.1 | ±0.69 | 0.23 |
| *pde2*Δ | 0.75 | ±0.58 | 0.19 |
| $WT^{HS}$ | 1.61 | ±0.66 | 0.22 |
| *pde2*$\Delta^{HS}$ | 3.04 | ±0.3 | 0.1 |

### Differences between heat-shocked samples were also apparent at the level of the proteome

Cellular proteins were isolated and separated using classical 2-D gel electrophoresis to determine the state of the yeast proteome after the heat shock, and compare it with that of the transcriptome. Twenty-three differentially produced protein spots (identified consistently on four independently run gels) were taken for MS and/or MS–MS identification of corresponding proteins (see Methods). Thirteen of them were unambiguously identified (Table 7). One of them, encoded by *TPI1*, was identified in two different spots, as shown by the presence of the multiple ions bearing the same mass : charge ratio. This was most likely a result of phosphorylation differences, as homologues of the same protein can migrate differently on the gel because of different pIs. It is known that phosphorylation decreases the pI of a protein. The proteins identified are amongst some of the highly abundant proteins in yeast (Ghaemmaghami *et al.*, 2003). Several additional spots were also characterized and found to represent degradation fragments of Wrs1p, Ssa1p (in two spots), and Tdh3p (in three spots), an observation which suggests that part of the cell proteins are degraded during the drastic heat shock from 30 °C to 50 °C applied in the present investigation. Interestingly, only one of these degradation products (a fragment of Wrs1p) was identified from the wild-type heat shock sample. The rest were all isolated from the mutant samples. Comparing the transcriptome data with the proteome results revealed that only some of the differentially produced proteins were encoded by genes that were accordingly differentially expressed. These included *ILV5, ENO1,*

**Table 7.** MS analysis of differentially produced proteins

| Protein | Encoding gene | Functional category (MIPS) | Isolated from |
|---|---|---|---|
| Acetohydroxy-acid isomeroreductase | *ILV5* (YLR355C) | Metabolism | $WT^{HS}$ |
| Ubiquitinol cytochrome *c* reductase chain B | *UCR2* (YPR191W) | Energy | $WT^{HS}$ |
| Enolase | *ENO1* (YGR254W) | Metabolism | $WT^{HS}$ |
| Tropomyosin | *TPM1* (YNL079C) | Cellular transport | $WT^{HS}$ |
| Thioredoxin reductase | *TRR1* (YDR353W) | Metabolism | $WT^{HS}$ |
| Outer mitochondrial membrane porin | *POR1* (YNL055C) | Cellular transport | $WT^{HS}$ |
| Histidine triad protein | *HNT1* (YDL125C) | Metabolism | $WT^{HS}$ |
| Carboxypeptidase Y inhibitor | *TFS1* (YLR178C) | Cell cycle and DNA processing | $WT^{HS}$ |
| Acid anhydride hydrolase pyrophosphate | *DCS1* (YLR270W) | Unclassified | $WT^{HS}$ |
| Inorganic pyrophosphatase | *IPP1* (YBR011C) | Unclassified | $WT^{HS}$ |
| Triosephosphate isomerase | *TPI1* (YDR050C) | Metabolism | *pde2*$\Delta^{HS}$ |
| Mitochondrial ribosomal protein | *MRP8* (YKL142W) | Protein synthesis | *pde2*$\Delta^{HS}$ |

Copyright © 2004 John Wiley & Sons, Ltd. 

*TPM1, TRR1, IPP1, TPI1* and *MRP8*, respectively. However, there was no correlation between the levels of gene and protein expression.

### Discussion

There are several reports on studies of the genomic expression responses to different stress conditions, including heat shock in the yeast *S. cerevisiae*. In all cases the heat shock entailed a temperature transition of 25 °C to 37 °C. Time-course experiments have shown that this results in changes in gene expression within minutes of the shock (Gasch *et al.*, 2000; Causton *et al.*, 2001). Adaptation of gene expression allows cells to survive sudden changes in the environment and provides the ability to resume normal growth upon return to more favourable conditions. Mutants with a constitutively active cAMP-dependent pathway, such as those arising from deletion of *PDE2*, or a constitutively activated *RAS2* allele ($RAS2^{Val19}$), are highly sensitive to a brief heat shock treatment at 50 °C (Sass *et al.*, 1986; Toda *et al.*, 1985). Following such a treatment, *pde2*Δ mutant cells lose viability, whilst a significant proportion, but not all of the wild-type cells, survive (Figure 1).

To our knowledge there have been no previous attempts to characterize the transcriptome and proteome of wild-type and *pde2*Δ cells subjected to a drastic heat shock. Our results suggest several factors, which appear to contribute towards survival upon such heat shock. Survival depends on the cell cycle phase, with cells in the budding phases ($S–G_2$, $G_2$ and $G_2–M$) being highly susceptible to high temperature heat shock. This effect could be due to differences in cAMP levels as cell cycle-mediated cAMP oscillation (about two-fold increase at the peak) has been reported for both glucose-limited and glucose-repressed synchronized cultures (Muller *et al.*, 2003); the highest cAMP levels were observed in cells in $S–G_2$ and $G_2–M$ cell cycle phases. The asynchronization in the original culture used in the shock experiment could explain the 50% survival in the wild-type cells.

Our study shows for the first time that the utilization of different carbon sources in the medium also has an effect on subsequent survival upon high-temperature heat shock. The *pde2*Δ mutant survived the heat shock to a greater extent if pre-grown on a non-fermentable carbon source such as glycerol (Figure 4). Constitutive activation of the cAMP-dependent pathway gives rise to high activity of PKA, leading to poor growth on non-fermentable and weakly fermentable carbon sources. It is therefore reasonable to assume that the *pde2*Δ mutant survives heat shock better after prior growth on a non-fermentable carbon source because the cells are not growing optimally and are, therefore, less susceptible to heat shock than the *pde2*Δ cells growing on glucose. The observation that the *pde2*Δ mutant shows downregulation in 11 of the 18 genes encoding hexose transporters (Andre, 1995; Kruckeberg, 1996; Nelissen *et al.*, 1997), combined with the fact that *MAL11* is also downregulated, could suggest an attempt to slow down growth in order to increase the chance of survival. Previously it has been shown that constitutive activation of the cAMP-dependent pathway inhibits maltose utilization by reducing both the transcription and activity of the maltose permease encoded by *MAL11* (Wanke *et al.*, 1997). Downregulation of this gene would be expected under normal conditions of growth; however, there is no evidence of this. Previously, no differential expression of the *MAL11* gene before heat shock was observed. The mechanisms which, during heat shock, increase the downregulation of the gene are at present unknown.

Heat stress has been reported to affect membrane-associated processes (Piper, 1995). Swan and Watson (1998) observed a relationship between increased ergosterol content and resistance to heat stress. The changes in ergosterol composition triggered by the heat shock suggest that the *pde2*Δ mutant is trying to utilize alternative methods to survive. This may be because it cannot induce the normal heat shock response due to the constitutive activation of the cAMP-dependent pathway causing negative feedback of the STRE-response elements.

There were several differentially produced proteins as revealed by the analysis of the proteome after heat shock. Protein degradation products were also identified and, with only one exception, the majority of them were in the mutant heat-shocked sample. Moreover, 10 of the 12 intact identified proteins were from the $WT^{HS}$ sample. As they corresponded to missing proteins from the mutant heat shock sample, it seems reasonable to speculate that they had been lost due to degradation. As degradation occurred predominantly in the mutant sample, it is highly unlikely that it was a result

Copyright © 2004 John Wiley & Sons, Ltd. 

of protein degradation during sample preparation. Rather, our study suggests either (a) yet another cause for the loss of viability in that a constitutively activated cAMP-dependent pathway determines a higher level of protein degradation during heat shock, or (b) that we could simply have a reflection of the lower viability of the mutant after heat shock.

All proteins that were unambiguously determined by MS, and/or tandem MS, were highly abundant. This is not unexpected, considering that they were visualized using colloidal blue staining, known to stain abundant proteins. Our analysis is therefore not representative of all the changes at the proteome level, which occur as a result of brief high-temperature heat shock. It only describes changes to the most abundant proteins. Further analysis by alternative and more sensitive methods are required to reveal all the changes affecting even the less abundant proteins in the proteome after heat shock. However, there was little if any correlation between transcriptome and proteome expression data.

### Acknowledgements

The work was supported by a COGEME grant, under the BBSRC 'Investigating Gene Function' Initiative awarded to SGO, SJG and LIS. DCH was supported by a Bioinformatics Research Fellowship from the MRC.

### References

Andre B. 1995. An overview of membrane transport proteins in *Saccharomyces cerevisiae*. *Yeast* **11**: 1575–1611.

Arkinstall SJ, Papasavvas SG, Payton MA. 1991. Yeast $\alpha$-mating factor receptor-linked G-protein signal transduction suppresses Ras-dependent activity. *FEBS Lett* **284**(1): 123–128.

Arthington-Skaggs BA, Jradi H, Desai T, Morrison CJ. 1999. Quantitation of ergosterol content: novel method for determination of fluconazole susceptibility of *Candida albicans*. *J Clin Microbiol* **37**(10): 3332–3337.

Baroni MD, Monti P, Alberghina L. 1994. Repression of growth-regulated $G_1$ cyclin expression by cyclic AMP in budding yeast. *Nature* **371**(6495): 339–342.

Bennet CB, Lewis LK, Karthikeyan G, *et al*. 2001. Genes required for ionizing radiation resistance in yeast. *Nature Genet* **29**(4): 426–434.

Boorstein WR, Craig EA. 1990. Transcriptional regulation of *SSA3*, an HSP70 gene from *Saccharomyces cerevisiae*. *Mol Cell Biol* **10**(6): 3262–3267.

Borges-Walmsley MI, Walmsley AR. 2000. cAMP signalling in pathogenic fungi: control of dimorphic switching and pathogenicity. *Trends Microbiol* **8**(3): 133–141.

Boy-Marcotte E, Tadi D, Perrot M, Boucherie H, Jacquet M. 1996. High cAMP levels antagonize the reprogramming of gene expression that occurs at the diauxic shift in *Saccharomyces cerevisiae*. *Microbiology* **142**: 459–467.

Broach JR. 1991. Ras-regulated signaling processes in *Saccharomyces cerevisiae*. *Curr Opin Genet Dev* **1**(3): 370–377.

Broach JR, Deschenes RJ. 1990. The function of *Ras* genes in *Saccharomyces cerevisiae*. *Adv Cancer Res* **54**: 79–139.

Cannon JF, Tatchell K. 1987. Characterization of *Saccharomyces cerevisiae* genes encoding subunits of cyclic AMP-dependent protein kinase. *Mol Cell Biol* **7**(8): 2653–2663.

Causton HC, Ren B, Koh SS, *et al*. 2001. Remodeling of yeast genome expression in response to environmental changes. *Mol Cell Biol* **12**(2): 323–337.

Drees BL, Sundin B, Brazeau E, *et al*. 2001. A protein interaction map for cell polarity development. *J Cell Biol* **154**(3): 549–571.

Dudoit S, Yang YH, Callow MJ, Speed TP. 2002. Statistical methods for identifying differentially expressed genes in replicated cDNA microarray experiments. *Statistica Sinica* **12**: 111–139.

Gasch AP, Huang M, Metzner S, *et al*. 2001. Genomic expression responses to DNA-damaging agents and the regulatory role of the yeast ATR homolog Mec1p. *Mol Cell Biol* **12**(10): 2987–3003.

Gasch AP, Spellman PT, Kao CM, *et al*. 2000. Genomic expression programs in the response of yeast cells to environmental changes. *Mol Cell Biol* **11**(12): 4241–4257.

Ghaemmaghami S, Huh WK, Bower K, *et al*. 2003. Global analysis of protein expression in yeast. *Nature* **425**(6959): 737–741.

Giaever G, Chu AM, Ni L, *et al*. 2002. Functional profiling of the *Saccharomyces cerevisiae* genome. *Nature* **418**(6896): 387–391.

Gimeno CJ, Ljungdahl PO, Styles CA, Fink GR. 1992. Unipolar cell divisions in the yeast *S. cerevisiae* lead to filamentous growth: regulation by starvation and *RAS*. *Cell* **68**(6): 1077–1090.

Hauser NC, Vingron M, Scheideler M, *et al*. 1998. Transcriptional profiling on all open reading frames of *Saccharomyces cerevisiae*. *Yeast* **14**(13): 1209–1221.

Hegde P, Qi R, Abernathy K, *et al*. 2000. A concise guide to cDNA microarray analysis. *Biotechniques* **29**(3): 548–550, 552–554, 556 passim.

Jones DL, Petty J, Hoyle DC, *et al*. 2003. Transcriptome profiling of a *Saccharomyces cerevisiae* mutant with a constitutively activated Ras/cAMP pathway. *Physiol Genom* **16**(1): 107–118.

Kruckeberg AL. 1996. The hexose transporter family of *Saccharomyces cerevisiae*. *Arch Microbiol* **166**: 283–292.

Kubota H, Sakaki Y, Ito T. 2000. $G_1$ domain-mediated association of the eukaryotic initiation factor $2\alpha$ kinase *GCN2* with its activator *GCN1* is required for general amino acid control in budding yeast. *J Biol Chem* **275**(27): 20 243–20 246.

Ma P, Wera S, Van Dijck P, Thevelein JM. 1999. The *PDE1*-encoded low-affinity phosphodiesterase in the yeast *Saccharomyces cerevisiae* has a specific function in controlling agonist-induced cAMP signalling. *Mol Biol Cell* **10**(1): 91–104.

Martinez-Pastor MT, Marchler G, Schuller C, *et al*. 1996. The *Saccharomyces cerevisiae* zinc finger proteins Msn2p and Msn4p are required for transcriptional induction through the stress response element (STRE). *EMBO J* **15**(9): 2227–2235.

Muller D, Exler S, Aguilera-Vazquez L, Guerrero-Martin E, Reuss M. 2003. Cyclic AMP mediates the cell cycle dynamics of

Copyright © 2004 John Wiley & Sons, Ltd. 

energy metabolism in *Saccharomyces cerevisiae*. *Yeast* **20**(4): 351–367.

Nelissen B, De Wachter R, Goffeau A. 1997. Classification of all putative permeases and other membrane plurispanners of the major facilitator superfamily encoded by the complete genome of *Saccharomyces cerevisiae*. *FEMS Microbiol Rev* **21**: 113–134.

Nikawa J, Sass P, Wigler M. 1987. Cloning and characterization of the low-affinity cyclic AMP phosphodiesterase gene of *Saccharomyces cerevisiae*. *Moll Cell Biol* **7**(10): 3629–3636.

Pan X, Heitman J. 1999. Cyclic AMP-dependent protein kinase regulates pseudohyphal differentiation in *Saccharomyces cerevisiae*. *Mol Cell Biol* **19**(7): 4874–4887.

Piper PW. 1995. The heat shock and ethanol stress responses of yeast exhibit extensive similarity and functional overlap. *FEMS Microbiol Lett* **134**: 121–127.

Reifenberger E, Boles E, Ciriacy M. 1997. Kinetic characterization of individual hexose transporters of *Saccharomyces cerevisiae* and their relation to the triggering mechanisms of glucose repression. *Eur J Biochem* **245**: 324–333.

Reifenberger E, Freidel K, Ciriacy M. 1995. Identification of novel HXT genes in *Saccharomyces cerevisiae* reveals the impact of individual hexose transporters on glycolytic flux. *Mol Microbiol* **16**: 157–167.

Ruis H, Schuller C. 1995. Stress signaling in yeast. *BioEssays* **17**(1): 959–965.

Sass P, Field J, Nikawa J, Toda T, Wigler M. 1986. Cloning and characterization of the high-affinity cAMP phosphodiesterase of *Saccharomyces cerevisiae*. *Proc Natl Acad Sci USA* **83**(24): 9303–9307.

Schmitt AP, McEntee K. 1996. Msn2p, a zinc finger DNA-binding protein, is the transcriptional activator of the multistress response in *Saccharomyces cerevisiae*. *Proc Natl Acad Sci USA* **93**(12): 5777–5782.

Sherman F, Fink GR, Hicks JB. 1986. *Methods in Yeast Genetics*. Cold Spring Harbor Laboratory Press: New York.

Shevchenko A, Wilm M, Vorm O, Mann M. 1996. Mass spectrometric sequencing of proteins silver-stained polyacrylamide gels. *Anal Chem* **68**(5): 850–858.

Steinmetz LM, Scharfe C, Deutschbauer AM, *et al*. 2002. Systematic screen for human disease genes in yeast. *Nature Genet* **31**(4): 400–404.

Suoranta K, Londesborough J. 1984. Purification of intact and nicked forms of a zinc-containing, $Mg^{2+}$-dependent, low $K_m$ cyclic AMP phosphodiesterase from bakers' yeast. *J Biol Chem* **259**(11): 6964–6971.

Swan TM, Watson K. 1998. Stress tolerance in a yeast sterol auxotroph: role of ergosterol, heat shock proteins and trehalose. *FEMS Microbiol Lett* **169**(1): 191–197.

Tatchell K. 1993. *RAS* genes in the budding yeast *Saccharomyces cerevisiae*. In *Signal Transduction in Prokaryotic and Simple Eukaryotic Systems*, Taylor BJ (ed.). Academic Press: London.

Thevelein JM. 1992. The RAS-adenylate cyclase pathway and cell cycle control in *Saccharomyces cerevisiae*. *Antonie Van Leeuwenhoek* **62**(1–2): 109–130.

Thevelein JM. 1994. Signal transduction in yeast. *Yeast* **10**(13): 1753–1790.

Thevelein JM, Cauwenberg L, Colombo S, *et al*. 2000. Nutrient-induced signal transduction through the protein kinase pathway and its role in the control of metabolism, stress resistance and growth in yeast. *Enz Microbial Technol* **26**(9–10): 819–825.

Thevelein JM, de Winde JH. 1999. Novel sensing mechanisms and targets for the cAMP-protein kinase A pathway in the yeast *Saccharomyces cerevisiae*. *Mol Microbiol* **33**(5): 904–918.

Toda T, Cameron S, Sass P, Zoller M, Wigler M. 1987a. Three different genes in *S. cerevisiae* encode the catalytic subunits of the cAMP-dependent protein kinase. *Cell* **50**(2): 277–287.

Toda T, Cameron S, Sass P, *et al*. 1987b. Cloning and characterization of *BCY1*, a locus encoding a regulatory subunit of the cyclic AMP-dependent protein kinase in *Saccharomyces cerevisiae*. *Mol Cell Biol* **7**(4): 1371–1377.

Toda T, Uno I, Ishikawa T, *et al*. 1985. In yeast, *RAS* proteins are controlling elements of adenylate cyclase. *Cell* **40**: 27–36.

Tokiwa G, Tyers M, Volpe T, Futcher B. 1994. Inhibition of $G_1$ cyclin activity by the Ras/cAMP pathway in yeast. *Nature* **371**(6495): 342–345.

Wanke V, Vavassori M, Thevelein JM, Tortora P, Vanoni M. 1997. Regulation of maltose utilization in *Saccharomyces cerevisiae* by genes of the *RAS*/protein kinase A pathway. *FEBS Lett* **402**: 251–255.

Wiederkehr A, Meier KD, Riezman H. 2001. Identification and characterization of *Saccharomyces cerevisiae* mutants defective in fluid-phase endocytosis. *Yeast* **18**(8): 759–773.

Wilson RB, Renault G, Jacquet M, Tatchell K. 1993. The *pde2* gene of *Saccharomyces cerevisiae* is allelic to *rca1* and encodes a phosphodiesterase which protects the cell from extracellular cAMP. *FEBS Lett* **325**(3): 191–195.

Wilson RB, Tatchell K. 1988. *SRA5* encodes the low-$K_m$ cyclic AMP phosphodiesterase of *Saccharomyces cerevisiae*. *Mol Cell Biol* **8**(1): 505–510.

Winston F, Dollard C, Ricupero-Hovasse SL. 1995. Construction of a set of convenient *Saccharomyces cerevisiae* strains that are isogenic to S288C. *Yeast* **11**(1): 53–55.

Xiao H, Lis JT. 1988. Germline transformation used to define key features of heat-shock response elements. *Science* **239**(4844): 1139–1142.

Yang YH, Dudoit S, Luu P, *et al*. 2002. Normalization for cDNA microarray data: a robust composite method addressing single and multiple slide systematic variation. *Nucleic Acids Res* **30**(4): e15.

Copyright © 2004 John Wiley & Sons, Ltd. 
